# Nipple-Sparing Mastectomy: Initial Experience Evaluating Patients Satisfaction and Oncological Safety in a Tertiary Care Centre in Jordan

**DOI:** 10.7759/cureus.19238

**Published:** 2021-11-03

**Authors:** Mohammad Athamnah, Nimah A Rabai, Zakaria W Shkoukani, Hussein S Al Azzam, Amer Abu-Shanab

**Affiliations:** 1 Department of General Surgery, Princess Basma Teaching Hospital, Irbid, JOR; 2 Department of General Surgery, Aberdeen Royal Infirmary, Scotland, GBR; 3 Office of Scientific Affairs and Research, King Hussein Cancer Center, Amman, JOR

**Keywords:** nipple sparing mastectomy, breast cancer, malignant phyllodes, ductal carcinoma in-situ, breast conservative surgery, primary breast malignancy

## Abstract

Introduction

Nipple-sparing mastectomy (NSM), a procedure involving careful dissection of the breast tissue whilst keeping the nipple-areola complex (NAC) intact, is now increasingly practiced amongst surgeons in the treatment of certain situations of breast cancer. Given the importance of breasts to the female body image, this type of conservative breast surgery takes into account patient satisfaction and overall cosmesis, whilst ensuring appropriate oncological safety.

Methods and results

Four nipple-sparing mastectomy procedures were performed in our tertiary care centre, Princess Basma Teaching Hospital, in Jordan between June and September 2019. Indications for these procedures included invasive ductal carcinoma, malignant phyllodes, and high-grade ductal carcinoma in-situ. Patients were carefully assessed prior to surgical intervention using radiological imaging, ensuring a distance from NAC of >2 cm in all cases. Procedures were performed successfully with minimal intraoperative and no post-operative complications. Follow-up was carried out at 24 months, with no cases of local or distant post-operative recurrence, and patient satisfaction was qualitatively measured with the use of a BREAST-Q questionnaire. The questionnaire demonstrated improved overall physical well-being and satisfaction with an average overall post-operative physical well-being of 97%.

Conclusion

Following nipple-sparing mastectomy and immediate submuscular reconstruction with silicone implants,patients demonstrated high levels of satisfaction and quality of life (QoL) as measured by BREAST-Q survey. Two years of follow-up confirmed high patient satisfaction with increased scores from the preoperative baseline level.

## Introduction

Conservative mastectomy, which includes skin-sparing and nipple-sparing mastectomy (NSM), is increasingly being advocated amongst surgeons as the gold standard for the treatment of specific situations of breast cancer [1]. As the name suggests, this type of surgery involves effective surgical removal of pre-cancerous and/or cancerous breast tissue, with particular attention to general cosmesis and body image [2]. Frequently, conservative mastectomy occurs in tandem with immediate reconstruction, which can further enhance overall patient satisfaction [3].

However, in hindrance to it becoming a mainstay in breast cancer treatment, conservative mastectomy has long been under scrutiny, especially so in oncological safety, as well as the risk of potential post-operative complications, most notably nipple necrosis [3]. Not only this, but also it’s relation to adjuvant and neo-adjuvant therapies has also been a topic of serious debate as of late. To introduce such conservative procedure in the local level, four surgeries were done on selective patients to study the oncological and satisfactory outcomes of such operations in a local level.

We herein report our hospital’s experience with nipple-sparing mastectomy procedures and evaluate the clinical outcome, patient post-operative cosmesis satisfaction, as well as cancer recurrence on follow-up.

## Materials and methods

Following approval by the Jordanian Ministry of Health Institutional Review Board (IRB), approval number Moh/REc/2021/96, a retrospective review study was carried out on four patients with varying breast pathologies whom after careful clinical assessment were found to be suitable for nipple-sparing mastectomy procedures. All surgeries included in this study were carried out during the period between June and September 2019.

On reviewing patient documentation, the following factors were taken into consideration: indications for surgery, pathologic/histologic findings, size of the tumor as well as distance from the nipple-areola complex (NAC), tumor laterality (uni- vs bilateral), post-operative complications, and follow-up. Pre-operative imaging was also reviewed and neoplastic involvement of the NAC (or a lack thereof) was noted.

The nipple-sparing mastectomy procedure involves dissection of the breast and removal of breast tissue, using radical incision, in an approach that effectively preserves the NAC, whilst proceeding to excise glandular tissue from within the nipple (differentiating NSM from a subcutaneous mastectomy). This was then followed by immediate reconstruction using submuscular silicone implants with/without mesh insertion.

Following review of patient documentation and necessary follow-up, patients were asked to attend the breast clinic, wherein they filled out a BREAST-Q version 2.0 reconstruction module on pre- and post-operative quality of life and level of satisfaction. This questionnaire was prepared by the University of British Columbia and consists of a series of questions related to psychosocial, sexual and physical well-being as well as pre- and post-operative satisfaction with breasts, nipple reconstruction, etc, with scales varying between 0-4 and 0-5. The results of these questionnaires were then aggregated onto an excel sheet, converted to percentages, and presented in a table. All patients were informed and consented prior to the inclusion of this data in our study [4].

## Results

A total of four nipple-sparing mastectomy procedures were performed in our hospital, three of which also underwent axillary lymph node dissection. The mean age of patients was 30 years (ranging between 19 and 45), and all four patients were medically free of any co-morbidities, none of which were smokers. 75% of tumours (3 out of 4) were located in the left breast. There were no cases of bilateral tumours in this study. The indications for all four mastectomy procedures included: invasive ductal carcinoma (not otherwise specified) in two patients (n=2), malignant phyllodes tumour (n=1), and high-grade ductal carcinoma in-situ (DCIS) with solid and comedo patterns (n=1). The above were determined by tru-cut biopsies pre-operatively.

All patients were evaluated pre-operatively using mammography, with one patient (n=1, phyllodes tumour) additionally undergoing an MRI. The phyllodes tumour case was found via imaging to have three lobulated masses; two of which were intraparenchymal, with the third being an sub-pectoral mass. The remainder of the cases were single neoplastic lesions. All four cases had tumours which on pre-operative imaging were found to be >2cm away from the NAC, and average tumour size was 4.3 cm.

No post-operative complications occurred in any of the four cases included in this study; however, one of the cases experienced significant intra-operative bleeding with a drop in Hb from 11 to 9 g/dL; this was treated conservatively.

Adjuvant chemotherapy alone was used in one (n=1) patient, while another patient (n=1, phyllodes tumour) received post-operative radiotherapy only [5]. Both adjuvant chemo and radiotherapy were used in one (n=1) patient. Post-operative histological analysis with effective DCIS removal concluded that the final patient (n=1) would not require adjuvant chemo nor radiotherapy.

All four patients were followed up after an average duration of 24 months, none of which showed any signs of local or distant disease recurrence.

**Figure 1:**
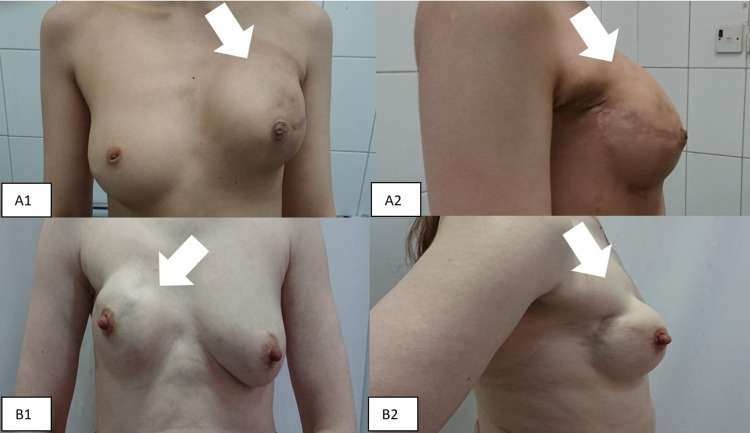
Post-operative pictures of two patients. A1: Left-sided NAC sparing mastectomy, frontal view; A2: Left-sided NAC sparing mastectomy, flipped side view; B1: Right-sided NAC sparing mastectomy, frontal view; B2: Left-sided NAC sparing mastectomy, side view. NAC: nipple-areola complex.

Following this, all four patients were asked to fill out a BREAST-Q version 2.0 pre- and post-operative quality of life (QoL) and satisfaction questionnaire. As can be seen in Table 1, the average overall psychosocial well-being was 88.5%, with sexual well-being averaging 87.5%. Average satisfaction with breast implants was 90.6%, while satisfaction with nipple reconstruction averaged 87.5%. Results regarding satisfaction with breasts were variable, with two patients reporting improved overall satisfaction post-operatively (81.3% to 88%, and 78% to 86.7% satisfaction), whilst the remaining two patients reported decreased satisfaction (93% to 83%, and 100% to 93% satisfaction). Physical well-being showed overall improvement amongst all patients with an average pre-operative well-being of 83%, and average post-operative well-being of 97%. All patients were satisfied with the surgeon and the medical team as a whole (100% satisfaction). Figure 1 shows the post-operative outcome for two patients, while the other two preferred not to be published.

**Table 1:** Results of BREAST-Q version 2.0 pre- and post-operative quality of life and satisfaction questionnaire.

	Patient no. 1	Patient no. 2	Patient no. 3	Patient no. 4
Psychosocial well-being	80%	96%	96%	82%
Sexual well-being	60%	90%	100%	100%
Physical well-being (pre-op)	70%	75%	98.5%	88%
Physical well-being (post-op)	100%	100%	100%	88%
Satisfaction with breasts (pre-op)	93%	81.3%	100%	78%
Satisfaction with breasts (post-op)	83%	88%	93%	86.7%
Satisfaction with implant	87.5%	100%	100%	75%
Satisfaction with nipple reconstruction	100%	75%	100%	75%
Satisfaction with surgeon	100%	100%	100%	100%

## Discussion

The management of early breast cancer has significantly improved over the last few decades. Current scientific developments no longer consider radical means of breast surgery an ideal option [6]. Conservative breast surgery (e.g., lumpectomy or quadrantectomy) allows the resection of disease alone with safety margins, without the need for total mastectomy. Following the introduction of conservative breast surgery was sentinel lymph node biopsy (SLNB), which effectively aided in avoiding unnecessary axillary clearances when lymph nodes are negative [7].

In certain cases, mastectomy cannot be avoided (e.g., small breasts not amenable to partial resections, large tumour size, multicentric disease). In such situations, conservative mastectomy (skin sparing mastectomy and nipple-sparing mastectomy) procedures can give more acceptable cosmetic outcomes and improve overall patient satisfaction; under the caveat that all criteria for conservative mastectomy apply [8,9]. Despite that, there were no local publications on the procedure making this article the first one that documents the procedure and its oncological and satisfactory outcomes in a local hospital.

Conservative mastectomy was first described by Freeman [10] in 1962 for the treatment of benign breast disease. Pennisi and Capozzi in 1989 reported a 10-year follow-up of 1,500 patients that underwent conservative mastectomy for high-risk lesions and benign disease and concluded that only 0.5% of operated patients developed breast cancer [11]. Furthermore, it was also found to be useful in cancer risk reduction for high-risk females [11-13]. Further studies regarding oncological safety came to show that conservative mastectomy was on par with modified radical mastectomy in cancer recurrence rates, and with time, the conservative approach has now gained popularity amongst surgeons for both prophylaxis and treatment of breast cancer [14].

Tumour size and distance between the tumour and NAC are important factors related to NAC involvement in malignancy. An increase in tumour size is commonly associated with increased incidence of NAC involvement - making nipple-sparing mastectomy a less favourable treatment option [15]. However, the tumour size cut-off point for NAC involvement is a matter of debate; with some studies [16] concluding that tumours smaller than 2 cm are less likely to involve the NAC. Other studies claimed tumours smaller than 3 cm to be more amenable to nipple-sparing mastectomy [17]. In this study the average tumour size was 4.3 cm, which is significantly more than previous studies have suggested as a cut-off, however in spite of this, no NAC involvement was found on imaging and no recurrence occurred at 24-month follow up, proving why this topic is currently debatable.

Another point of debate is the safe distance between the tumour and NAC, with few studies claiming a distance of more than 2.5 cm makes NAC involvement extremely rare [18], while others claim a distance of less than 2 cm to involve malignancy of the NAC in 50% of cases [16]. Tumour distance from the NAC in this study was >2 cm in all cases, with no recurrences, thus supporting the above.

Some complications reported by previous studies include surgical site infection, mastectomy skin flap necrosis, NAC necrosis, seroma formation, haematoma, and silicone implant/expander loss [19]. Early studies on nipple-sparing mastectomy reported high rates of NAC necrosis due to decreased blood supply and ischaemia [1,20-21], while other studies showed that complication rates decreased in nipple-sparing mastectomy and that this procedure was in fact more favourable and complication-free [19,22]. The patients included in this case study were medically fit with no significant past medical history and no history of smoking - thus decreasing the risk of possible complications. This perhaps explains the reason no complications were found in any of the subjects in this study.

Traditionally, less aggressive breast cancer surgery improved cosmetic outcome without compromising oncological safety. Concerns regarding oncological safety of conservative breast surgery (lumpectomy or quadrantectomy) were soon reassured when Veronesi and colleagues published a randomised control trial regarding this matter [23].

Similarly, conservative mastectomy is now under scrutiny as to whether it is equally effective with the same margin of safety as the more invasive counterparts. Multiple studies [1,24-26] have investigated this matter in depth and local recurrence rates following prolonged follow-up varied anywhere between 1% and 12%; however, all studies concluded that nipple-sparing mastectomy is not associated with any adverse oncological outcomes when compared to other mastectomies for the treatment of breast cancer [27]. It is worth noting that there were no incidents of recurrence in the 4 cases included in our case study following nipple-sparing mastectomy procedures after 24-month follow-up.

Since patient satisfaction and body image are of paramount importance in conservative mastectomy procedures including nipple-sparing mastectomies, it is important to evaluate patients’ pre-operative psychosocial, sexual and physical well-being as well as general body image satisfaction, to allow for a re-assessment on post-operative follow-up. In doing so, this gives an indication as to whether the procedure is achieving one of its main purposes or not. BREAST-Q questionnaires were distributed to the patients in this study on post-operative follow-ups after 24 months of their surgeries, in an attempt to quantify the degree of satisfaction and whether any improvement in quality of life was witnessed following surgery. As Table 1 demonstrates and as described in the results section above, the patients in this case study reported high levels of psychosocial and sexual well-being, were considerably satisfied with breast silicone implants as well as nipple reconstruction, and demonstrated a significant improvement in overall physical well-being following surgery. Satisfaction with breasts showed conflicting evidence, as two patients showed an improvement in satisfaction, whilst the other two patients reported the opposite. Those that reported decreased satisfaction were dissatisfied with the appearance, contour, or hardness of the breast tissue. Overall, the results of this questionnaire are thus in accordance with studies of a similar nature, demonstrating improved patient satisfaction and overall body image [28-30].

Given the aforementioned findings, and when taking into consideration the limited sample size, further studies would be recommended with larger study samples to further investigate the effectiveness of NSM as a means of managing specific types of breast cancer when it is oncologically safe to do so. Furthermore, the role of neoadjuvant and adjuvant radiotherapy and chemotherapy in conjunction with NSM procedures requires further investigation.

## Conclusions

Despite considerable cosmetic and psychological benefits associated with nipple-sparing mastectomy, there has been a reluctance to adopting the procedure due to concerns of higher recurrence rates and increased intra- and post-operative complications. In spite of this, proper selection of patients based on rigorous criteria can result in satisfactory cosmetic outcomes, improved physical, psychosocial and sexual well-being, alongside acceptable oncological control.
